# The effects of deep-brain non-stimulation in severe obsessive-compulsive disorder: an individual patient data meta-analysis

**DOI:** 10.1038/s41398-019-0522-6

**Published:** 2019-08-05

**Authors:** Koen Schruers, Samantha Baldi, Tijl van den Heuvel, Liesbet Goossens, Laura Luyten, Albert. F. G. Leentjens, Linda Ackermans, Yasin Temel, Wolfgang Viechtbauer

**Affiliations:** 1 Department of Psychiatry and Neuropsychology, Maastricht University Medical Center, Maastricht, The Netherlands; 2 Center for Psychology of Learning and Experimental Psychopathology, Faculty of Psychology and Educational Sciences, KU Leuven, Leuven, Belgium; 3 Department of Neurosurgery, Maastricht University Medical Center, Maastricht, The Netherlands

**Keywords:** Psychiatric disorders, Scientific community

## Abstract

Non-intervention-related effects have long been recognized in an array of medical interventions, to which surgical procedures like deep-brain stimulation are no exception. While the existence of placebo and micro-lesion effects has been convincingly demonstrated in DBS for major depression and Parkinson’s disease, systematic investigations for obsessive-compulsive disorder (OCD) are currently lacking. We therefore undertook an individual patient data meta-analysis with the aim of quantifying the effect of DBS for severe, treatment-resistant OCD that is not due to the electrical stimulation of brain tissue. The MEDLINE/PubMed database was searched for double-blind, sham-controlled randomized clinical trials published in English between 1998 and 2018. Individual patient data was obtained from the original authors and combined in a meta-analysis. We assessed differences from baseline in obsessive-compulsive symptoms following sham treatment, as measured by the Yale-Brown Obsessive-Compulsive Scale (Y-BOCS). Four studies met the inclusion criteria, randomizing 49 patients to two periods of active or sham stimulation. To preclude confounding by period effects, our estimate was based only on data from those patients who underwent sham stimulation first (*n* = 24). We found that sham stimulation induced a significant change in the Y-BOCS score (*t* = −3.15, *P* < 0.005), lowering it by 4.9 ± 1.6 points [95% CI = (−8.0, –1.8)]. We conclude that non-stimulation-related effects of DBS exist also in OCD. The identification of the factors determining the magnitude and occurrence of these effects will help to design strategies that will ultimately lead to a betterment of future randomized clinical trials.

Obsessive-compulsive disorder (OCD) is a highly debilitating neuropsychiatric disorder, affecting around 2% of the population. It is characterized by repeated intrusive thoughts, images or impulses (i.e., obsessions) that cause negative emotion (usually labeled as anxiety) and trigger behaviors aimed at reducing this negative affect (i.e., compulsions)^1^. Effective treatment is available in the form of cognitive behavioral therapy (mainly exposure therapy) and pharmacological treatment (mainly with serotonin reuptake inhibitors (SSRIs) and clomipramine)^1^. In spite of these treatment options, an estimated 10–20% of affected individuals remains resistant to all therapies, suffers from severe incapacitating symptoms and, consequently, maintains a very low quality of life^2^. For this group of patients, the possibility of deep-brain stimulation was introduced in 1999, being regarded as an appealing “last resort option” mainly due to its adjustability and reversibility^3^. By delivering electrical current to specific locations in the brain, DBS therapy can be tailored to the individual patient’s level of complaints, and most stimulation-induced side effects can be minimized by adjusting stimulation parameters^2^. The precise mechanism of action of DBS is only partially known, with evidence showing that DBS can exert its effect through both electrical activation and inhibition of brain areas and circuits that are involved in the pathophysiology of OCD^4-6^. Alongside numerous uncontrolled case reports, series, and trials^7-11^, several blinded, randomized controlled evaluations have demonstrated its effectiveness^12-17^, using different targets in the brain and leading to US Food and Drug Administration (FDA) and Conformité Européene (CE) mark approval through a humanitarian device exemption in 2009^18,19^. A recent meta-analysis reported a response percentage of 60% and a global reduction in OCD symptoms of 45%, along with considerable yet not systematically assessed improvement in some aspects of quality of life^20^.

However, as with all treatments in medicine, there is the need to discriminate between the therapeutic benefit due to the intervention “per se” and that due to other inherently related factors. It has long been recognized that the role of placebo responses is to be taken into account, as the simple act of receiving any treatment can be efficacious by itself and induce clinically meaningful neurobiological changes in an array of human health-related conditions^21^. Neurosurgical procedures are no exception to this rule, as was elegantly demonstrated in trials of DBS for Parkinson’s disease (PD)^22,23^. These findings confirm the notion that surgical procedures do include a placebo component, mainly mediated by the expectation of benefit that is inherently triggered in the patient. However, obvious ethical issues, mainly about the inclusion of sham surgery, hinder the exploration of the role of placebo in surgical treatments: a sham surgical procedure that includes administration of anesthetic drugs and inflicting tissue damage is inherently dangerous and therefore generally deemed unacceptable^24,25^.

DBS may be an exception to this rule, as the surgical procedure per se is not intended to cause any benefit, but it is merely the vehicle for the therapeutic effect of electrical current on brain cells and circuits. In the attempt to control for placebo responses, several studies adopted randomized, blinded crossover designs, in which patients are randomly assigned to either real (ON) or sham (OFF) stimulation for several weeks, and then switched to the other condition in the second part of the study. However, this approach is not without problems. So-called period effects and carryover effects are frequent yet not systematically assessed confounding factors characteristic of this study design. Period effects occur when the effect of stimulation differs between the ON–OFF group and the OFF–ON group^26^. Carryover effects refer to the possibility that the effect of the intervention provided in the first period extends into the second intervention period, a risk that is ideally minimized by an appropriately long washout between the different intervention arms^26^. Furthermore, it is well-documented that some of the effects and side effects of DBS occur very rapidly^27^, thus possibly giving rise to problems with blinding, especially during the second period of the study. Despite these issues, crossover designs in the context of DBS surgery are valuable ways of accounting for potential placebo effects, as they control for information bias and address the afore-mentioned ethical concerns of insertion or non-insertion of the device itself^28^.

To date, there have been several narrative reviews, systematic reviews and meta-analyses summarizing existing data on the effectiveness and safety of DBS surgery in OCD^20^^,^^28–30^. However, none of them explicitely focused on the magnitude of non-stimulation-related effects. This knowledge is not only necessary to design better clinical trials, but can also inform clinical practice on which other elements of the treatment context might be harnessed to reinforce patient’s response and motivation. We therefore undertook an individual patient data meta-analysis with the aim of quantifying the effect of DBS for severe, treatment-resistant OCD that is not due to the electrical stimulation of brain tissue. We included all double-blind, sham-controlled randomized clinical trials (RCTs) and looked at changes in clinical symptoms following a period of sham stimulation, while controlling for the occurrence of period effects. Our hypothesis was that a statistically significant non-stimulation effect would exist.

## Methods

### Search strategy and study selection

Studies were identified by searching electronic databases and scanning reference lists of relevant papers and reviews. Searches were restricted to human studies published between 1998 and 2018. This search was applied to the MEDLINE/PubMed database using the following Mesh terms: “Obsessive-Compulsive Disorder” AND (“Deep Brain Stimulation” OR “Electric Stimulation”). Titles and abstracts of all papers identified in the electronic searches were inspected to identify clinical studies on DBS in OCD. The full-text of candidate studies was then obtained to screen for relevance and eligibility.

Studies had to meet the following criteria in order to be included: (1) use of a randomized, double-blind, sham-controlled design; (2) reporting of baseline and post-sham (i.e., post-implantation after receiving sham stimulation) treatment scores on the Yale-Brown Obsessive-Compulsive Scale (Y-BOCS); (3) use of unique data; (4) availability of individual patient data; (5) published in English in a peer-reviewed journal. Thus, posters, conference abstracts, case reports, letter to editors and commentaries were discarded. Reference lists of narrative and systematic reviews and meta-analyses were inspected for additional potential sources. Crossover designs consisting of multiple, not-consecutive ON vs. OFF stimulation blocks were excluded.

### Data extraction and quality assessment

Information was extracted from each included trial on: (1) patient characteristics (including age, gender, illness duration, and severity); (2) design characteristics (including duration of optimization phase following surgery, duration of stimulation arms, presence and duration of a washout period); (3) surgical target for electrode implantation; (4) outcome measures and (5) number of dropouts.

The primary outcome was the effect of sham stimulation on OCD symptoms according to changes in the Y-BOCS scores. The Y-BOCS is the most widely used, validated OCD rating scale^31^, with scores ranging from 0 (no symptoms) to 40 (extremely severe OCD symptoms). Individual patient data was used instead of pooled averages. In studies where Y-BOCS scores were grouped per condition, individual data was retrieved by contacting the original authors. All but one responded and provided the raw data that was then used for the analysis.

Each study was independently evaluated to ascertain the validity of the included RCTs. According to the Cochrane methods, the risk of bias was categorized as high, low, or unclear on the adequacy of randomization, concealment of allocation and blinding of participants, personnel and outcome assessors.

Eligibility assessment, study selection and data extraction were performed by three independent researchers (S.B, L.G., and T.vd. H.), and all discrepancies were resolved by re-checking and further discussing source papers between reviewers.

### Statistical analysis

Analyses were conducted using R^32^. As our primary outcome measure, we computed individual change scores by subtracting the baseline Y-BOCS from the post-sham Y-BOCS score. In order to ensure the highest possible level of homogeneity of the data, baseline was defined as the last Y-BOCS measurement before implantation. We first evaluated the relationship between the baseline and post-sham Y-BOCS score by computing Pearson’s correlation and intraclass correlation (ICC) estimates separately for the two order conditions. The R packages *lme4*^33^ and *lmerTest*^34^ were then used to perform linear mixed effects analyses. We tested for period effects (OFF–ON vs. ON–OFF) by including data from both periods and entering order of stimulation arms as a fixed effect into the model. Random intercepts for the included studies were added to the model. Should an order effect be detected, the analysis was confined to data derived from the first crossover period, thus including only those patients who underwent sham stimulation first (i.e., OFF–ON). *P*-values of the fixed effects were determined using the Satterthwaite’s degrees of freedom method, as implemented in *lmerTest*’s ANOVA function. This approach was chosen because it was shown to produce more acceptable Type I error rates even for smaller sample sizes as compared to more commonly used methods for evaluating significance such as likelihood ratio tests or the t-as-z approach^35^.

## Results

### Study characteristics

**Fig. 1:**
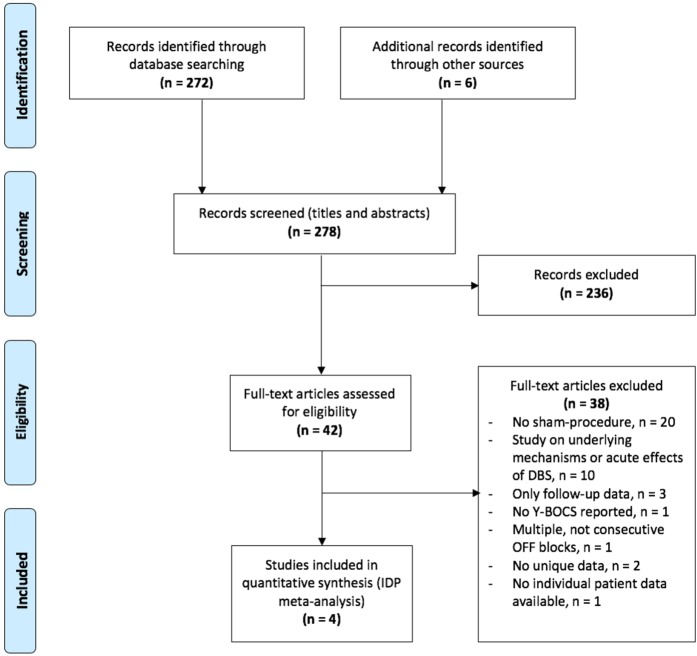
**Flow of information according to PRISMA statement, study selection and reasons for exclusion**

In the initial electronic searches, 272 studies of interest were found. Snowball searching of reference lists of relevant papers and reviews identified an extra six studies, yielding a total of 278 records of which titles and abstracts were screened (Fig. 1). Of these, 42 full-text articles were deemed potentially relevant and were assessed for eligibility. Thirty-eight studies were excluded, mainly because they did not include a sham procedure (*n* = 20), or focused on outcomes or effects of DBS that differed from those of interest for the present study (*n* = 10). Others only included follow-up data (*n* = 3), failed to report Y-BOCS scores (*n* = 1) or did not present unique data (*n* = 2). One of the excluded studies was a double-blind, sham-controlled trial, in which patients were randomized to multiple, separate periods of active vs. sham stimulation. However, the exact order of stimulation arms was not reported for all patients^12^. A last eligible study was excluded because the authors did not react to our request to provide the individual data^14^.

Four studies were finally included in the analysis, all of them being double-blind, sham-controlled RCTs assessing the efficacy of DBS for primary, treatment-resistant OCD^13,15-17^. The included studies provided data for a total of 49 patients, who received DBS in the subthalamic nucleus^13^, the nucleus accumbens^15^, the anterior limb of the internal capsule^16,17^, or the bed nucleus of the stria terminalis^17^. All studies included patients aged between 18 and 65 years, suffering from severe OCD (i.e., Y-BOCS score of at least 25) for at least 5 years and meeting stringent criteria for refractoriness to treatment^36^. Table 1 summarizes the main design characteristics of the included studies. Three comprised an open, exploratory period for optimization of the stimulation parameters before entering the double-blind randomized phase^13,16,17^. Three studies adopted a crossover design, with duration of the stimulation arms of 3 months^13,15,17^. Of these, only one study applied a washout period lasting 1 month^13^, whereas in the others the crossover to the second stimulation condition happened consecutively. One of the included studies adopted a staggered-onset design^16^. That is, at 30-days post-implantation, half of the patients were assigned to sham DBS for 30-days prior to active-DBS (OFF–ON), while the others were assigned to active-DBS straightaway (ON–ON).

**Table 1:** Study characteristics

Authors	No. of patients	Age mean (SD)	DBS target	Design	Optimization phase	Arm duration	Wash-out period	Drop-out
Mallet et al.^13^	17	43.05 ( ± 7.9)	STN	Crossover	3 months	Two 3-months periods	1 month	1
Goodman et al.^16^	6	36.2 ( ± 8.6)	ALIC	Staggered-onset	30 days	Two 30-days periods (OFF–ON) vs. 60 days (ON–ON)	No	0
Huff et al.^15^	10	36.3 ( ± 6.4)	Right NAc	Crossover	No	Two 3-months periods	No	0
Luyten et al.^17^	17	38.7 ( ± 10.9)	ALIC/BNST	Crossover	9 months (average)	Two 3-months periods^a^	No	0

*STN* subthalamic nucleus, *NAc* nucleus accumbens, *ALIC* anterior limb of the internal capsule, *BNST* bed nucleus of the stria terminalis

^a^An escape procedure was established in case of unbearable worsening of symptoms during the blinded phase. Median duration of the ON phase (89 days) was longer than that of the OFF phase (44 days)

In all studies, allocation to active and sham treatment was determined by randomization, albeit only one adequately reported on the method used for sequence generation and allocation concealment^13^. Blinding of assessors and patients was preserved at least until the end of the crossover phase in all studies. However, none of them reported on formal testing on the effectiveness of blinding, and only three studies at least addressed the issue in their discussion^13,15,17^. Whereas unblinding might have been prevented by the use of either fixed^15^ or below the side-effects threshold^13^ stimulation parameters, this might have not been the case when a relatively extensive optimization phase was performed^16,17^.

### Post-sham stimulation outcome evaluation

Individual patient data was available for all four trials, randomizing 49 patients to two periods of either active or sham stimulation. Data from three patients who did not undergo a period OFF stimulation were excluded from the analysis^16^.

We first investigated the occurrence of period effects by assessing the relationship between baseline and post-sham Y-BOCS score separately for the two order conditions. Graphical exploration of this relationship showed larger drops in Y-BOCS score for the OFF–ON (mean = −4.91, SD = 7.65) as compared to the ON–OFF condition (mean = −1.77, SD = 5.38) (Supplementary Fig. 1). This was also reflected by larger Pearson’s correlation and intraclass correlation (ICC) estimates for the ON–OFF condition (*r* = 0.50, ICC = 0.45) than for the OFF–ON condition (*r* = 0.39, ICC = 0.07).

We then formally tested for period effects by fitting a linear mixed effects model to data from both periods (*n* = 46). Visual inspection of residual plots did not reveal any conspicuous deviations from homoscedasticity or normality. Following a period OFF-stimulation, the Y-BOCS score lowered by 4.52 ± 1.73 [95% CI = (−7.90, −0.67)], constituting a marginally significant change (*t* = −2.60, *p* = 0.0580). The order of stimulation arm did not have a significant influence on Y-BOCS score reduction (*b* = 3.46, SE = 1.91, *t* = 1.80, *p* = 0.078).

**Fig. 2:**
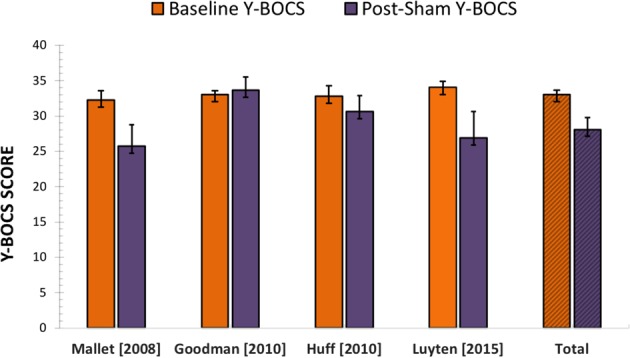
**Mean Y-BOCS scores at baseline and post-sham stimulation with their respective standard error of the mean (SEM), plotted for the included studies and the pooled individual data (*n*** **=** **24).** Only data of patients who entered the sham condition first is shown

However, in light of the relatively small sample size, the relatively large size of the coefficient for the period effect and the above-mentioned risk of unblinding, we decided to base our estimate on the data from the OFF–ON condition only (*n* = 24). The mean Y-BOCS scores at baseline and post-sham were respectively 33 (SD = 3) and 28 (SD = 8) points (Fig. 2). The mixed effects model fitted to these data showed that sham stimulation induced a significant change in the Y-BOCS score (*t* = −3.15, *p* = 0.0045), lowering it by 4.91 ± 1.56 points [95% CI = (−8.03, −1.79)].

## Discussion

The present study shows, using individual patient data from published randomized controlled trials, that DBS for treatment-refractory OCD involves a statistically significant “non-stimulation” effect, thus confirming our initial hypothesis. The data are suggestive of a period effect: albeit not statistically significant, the recorded smaller drops in Y-BOCS score in the ON–OFF condition might be suggestive of ineffective blinding, with patients being more or less aware of the clinical effects of active stimulation. Thus, in the attempt of reducing the risk of underestimating the magnitude of a non-stimulation-related effect, we decided to base our estimate on data from the OFF–ON condition only. The average difference in Y-BOCS score between baseline and post-sham stimulation amounted to about 5 points or 14.9%, which constitutes a meaningful difference in clinical terms^37^.

To our knowledge, this is the first study to explicitly examine the non-stimulation-related effects of DBS in OCD. There are however some studies exploring the clinical effect of expectation in DBS of the subthalamic nucleus (STN) for Parkinson’s disease that can be informative here. Two of those reported that blinded assessment of treatment was associated with a smaller clinical effect compared to unblinded assessment^23,38^. In a third study, a positive versus negative expectation bias was purposely induced. It was found that hand movement following STN stimulation was faster when patients expected good motor performance than when they did not^39^. Finally, in a post-hoc analysis of a large crossover DBS study for Parkinson’s disease^40^, it was stated that patients who first entered the OFF condition showed better response to active-DBS than vice versa^41^ (but see ref. ^42^). Altogether, these studies clearly indicate that expectations can influence the effects of DBS.

Regarding the magnitude of this effect and its clinical significance in psychiatric disorders, the “Reclaim”^43^ and “Broaden”^44^ DBS trials for depression can be informative, as in both these studies attention was given to a possible placebo effect from the outset. In the “Reclaim” trial, the response rate in the sham group was estimated at 15% and turned out to be 14.3%. In the “Broaden” study these respective figures were 18.5% and 17%. Although OCD is suggested to be less prone to placebo effects than depression^45^, these numbers are in line with the findings of the present study.

Several factors may influence the occurrence and magnitude of expectation effects. First, the length of the post-surgery optimization phase might play a role. It can be hypothesized that the less time between lead placement and start of the blinded crossover phase, the stronger the expectation may be, reflecting the hope patients might have for improvement through a treatment applied in the forseeable future. On the other hand, a long period with elaborate testing of the ideal study parameters is likely to give patients the chance to figure out what effects (wanted or unwanted) are associated with the stimulation being “ON”, thereby increasing the risk of effectively unblinding the period after randomization^25^. Similarly, it might be the case that placebo responses could be smaller for long periods of sham stimulation. Although not explored in the context of DBS, continuation studies in clinical trials of antidepressants indeed suggest that placebo effects are not sustained long-term, demonstrating the superiority of continued medication over prolonged placebo treatment in preventing symptom reoccurrence^46^. Another factor that may be tightly related to expectation effects is the probability of receiving the active treatment. When analyzing clinical outcomes of DBS in PD patients over the course of 6 months, Goetz et al.^47^ reported increased odds of placebo responses in trials where the probability of receiving the active treatment was 50% compared to when the probability was lower. A subsequent study from Lidstone et al.^48^ registered placebo responses specifically when the stated probability of receiving active medication was 75% but not when it was lower, thus demonstrating the capacity of verbal instructions to modulate clinical effects. This has important implications for the design of clinical trials, in that the magnitude of expectation effects could be monitored by manipulating the quality of the information given to the patient, e.g., in regards to the probability of receiving active-DBS.

Closed-loop stimulation may provide a possible way forward by reducing the expectation-induced therapeutic benefit. Closed-loop DBS uses a brain-computer interface that provides stimulation upon detection of an abnormal state in the brain, thereby rendering the procedure adaptable to disease fluctuations without the patient being explicitly aware of its functioning^49^. Valuable biomarkers and tangible therapeutic effects have been found for neurological conditions such as epilepsy and Parkinson’s disease^49^. Recent advances in the development of closed-loop devices for the treatment of refractory depression^50^ make this a promising avenue for the treatment of psychiatric conditions as well. However, before closed-loop stimulation can be considered a concrete option, a great deal of effort first has to be devoted to the identification of reliable and independent biomarkers for such a complex disease as OCD. Some promising steps have already been taken in this direction in rats^51^ as well as patients^52^.

Apart from expectation, non-stimulation-related effects also include the so-called “micro-lesion” effect: the placement of the DBS lead with a section surface of around one square millimeter causes a small lesion that can have a clinical effect in itself. Stereotactic lesions have been used for decades in the treatment of severe OCD. Although no direct head-to-head comparison studies are available, their efficacy seems roughly equivalent to treatment with DBS^53^. Lesions that are made with therapeutic aim typically have a volume of several cubic millimeters^54,55^, and are therefore considerably larger than those made by the placement of a DBS lead. Nevertheless, a therapeutic effect of such a small lesion cannot be excluded, especially given the relatively small volume of the targeted anatomical structures in OCD (STN: varies between 180–720 mm^3^, NAc: 433 ± 100 mm^3^, BNST: 190 mm^3^). The matter has not been investigated in OCD, but studies in Parkinson’s disease convincingly showed the existence of a micro-lesion effect on motor symptoms such as tremor, rigidity, and bradykinesia^56-58^, as well as on cognitive functions or on emotion recognition^59-61^. Interestingly, several of those studies report that the micro-lesion effect occurs immediately and is detectable until 6 or even 12 months after surgery, indicating that a transitory effect is highly unlikely.

Although systematic investigations are yet to be conducted, the existence of this effect in OCD cannot be ruled out given the indications from the above studies in Parkinson’s disease and the history of lesion studies in OCD. In order to design clinical trials that can profitably evaluate DBS efficacy, the magnitude and occurrence of micro-lesion effects are to be pinpointed independently from the effects induced by the expectation of benefit. Having been defined as the “highest quality prospective data available on the lesion effect in PD”^25^, the study by Okun et al.^57^ can provide useful pointers in this regard. In this open-label trial, 25% of patients were activated during the first 3 months, while the rest remained without activation constituting a “pure lesion group”. If patients are indeed explicitly aware that the stimulation is OFF, then the expectation of a benefit is likely reduced to the minimum or even eliminated, and eventual improvements in symptoms could be ascribed to the micro-lesion only. It is also suggested that findings of possible micro-lesion effects at 6 months post-implantation are not necessarily an index of persistence, but could also be due to insufficiently long medication or stimulation washouts before testing^25^. Thus, follow-ups at >3 months could be done to assess the duration of these effects by ensuring sufficiently long washout periods.

The present results must be considered and interpreted within the framework of some limitations. Our study pooled individual patient data from existing RCTs to obtain an estimate of non-stimulation-related effects in DBS trials. This approach might be questionable, given that some design characteristics differed across studies. However, statistical analysis with mixed modeling demonstrated that only a small portion of the variance was explained by between-study differences, which likely did not introduce substantial heterogeneity. Thus, we believe that pooling data of different studies allowed us to estimate the effect of interest with more precision than is possible in a single study, even more so when facing the issue of small sample sizes. Caution must be taken in the interpretation of the results also in light of the varying quality of the included studies, especially in regards to blinding of participants. Potential unblinding might indeed have occurred in some studies due to the use of a prolonged post-surgery optimization phase. Finally, we limited our analysis to RCTs published in English in peer-reviewed journals, thus possibly introducing a publication and language bias.

In conclusion, non-stimulation-related effects of DBS do exist also in OCD, and need to be addressed in future clinical trials. A careful evaluation and handling of variables like verbal instructions, allocation type (masked vs. unmasked) and length of the optimization phase will allow an informed management of expectation-induced effects. Concurrently, studies aiming to pinpoint the magnitude and duration of micro-lesion effects will progressively lead to the betterment of randomized controlled trials, which will then succeed in disentangling stimulation-related therapeutic benefit from that due to non-stimulation-related factors.

## Supplementary information

Supplementary Appendix
