# Supplementary material for: The effects of deep-brain non-stimulation in severe obsessive-compulsive disorder: an individual patient data meta-analysis
Source: Transl Psychiatry. 2019 Aug 5;9:183. doi: 10.1038/s41398-019-0522-6 (PMC6683131; doi:10.1038/s41398-019-0522-6)
Supplement: Supplementary file 1 — Supplementary Appendix [file 41398_2019_522_MOESM1_ESM.docx]

**Supplementary Appendix**

1

***“The Effects of Deep Brain Non-Stimulation in Severe Obsessive-Compulsive
Disorder: an Individual Patient Data Meta-Analysis”***

**Koen Schruers, Samantha Baldi, Tijl van den Heuvel, Liesbet Goossens, Laura Luyten, Albert
Leentjens, Linda Ackermans, Yasin Temel, Wolfgang Viechtbauer**

**Supplementary Figures**

**
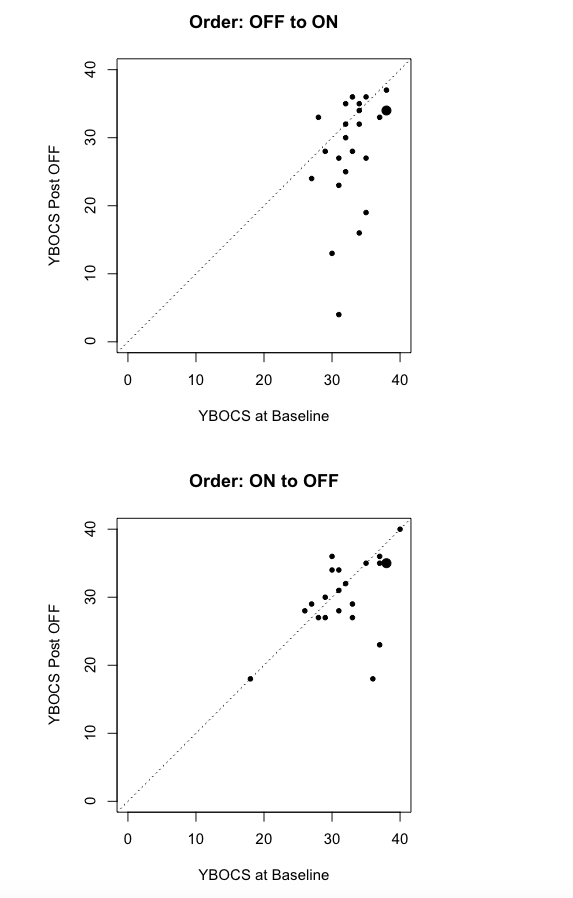
**

**Supplementary Figure 1.** Scatterplots of baseline Y-BOCS score against Y-BOCS score post-sham stimulation for the two order conditions: OFF-ON (n = 24) vs. ON-OFF (n = 22).
